# Current insights and future directions of LncRNA Morrbid in disease pathogenesis

**DOI:** 10.1016/j.heliyon.2024.e36681

**Published:** 2024-08-22

**Authors:** Haiqiong Yang, Jiali Gao, Zaiyong Zheng, Yang Yu, Chunxiang Zhang

**Affiliations:** a Department of Cardiology, The Affiliated Hospital, Southwest Medical University, Luzhou, China; b Key Laboratory of Medical Electrophysiology, Ministry of Education & Medical Electrophysiological Key Laboratory of Sichuan Province, (Collaborative Innovation Center for Prevention of Cardiovascular Diseases), Institute of Cardiovascular Research, Southwest Medical University, Luzhou, China; c School of Pharmacy, Southwest Medical University, Luzhou, China; d Department of pharmacy, Luzhou people's hospital, Luzhou, China

**Keywords:** Long non-coding RNA, LncRNA Morrbid, MIR4435-2HG, Tumor, Cardiovascular disease

## Abstract

Non-coding RNAs have emerged as important regulators of gene expression and contributors to many diseases. LncRNA Morrbid, a long non-coding RNA, has been widely studied in recent years. Current literature reports that lncRNA Morrbid is involved in various diseases such as tumors, cardiovascular diseases, inflammatory diseases and metabolic disorder. However, controversial conclusions exist in current studies. As a potential therapeutic target, it is necessary to comprehensively review the current evidence. In this work, we carefully review the literature on Morrbid and discuss each of the hot topics related to lncRNA Morrbid.

## 1 Introduction

**Fig. 1:**
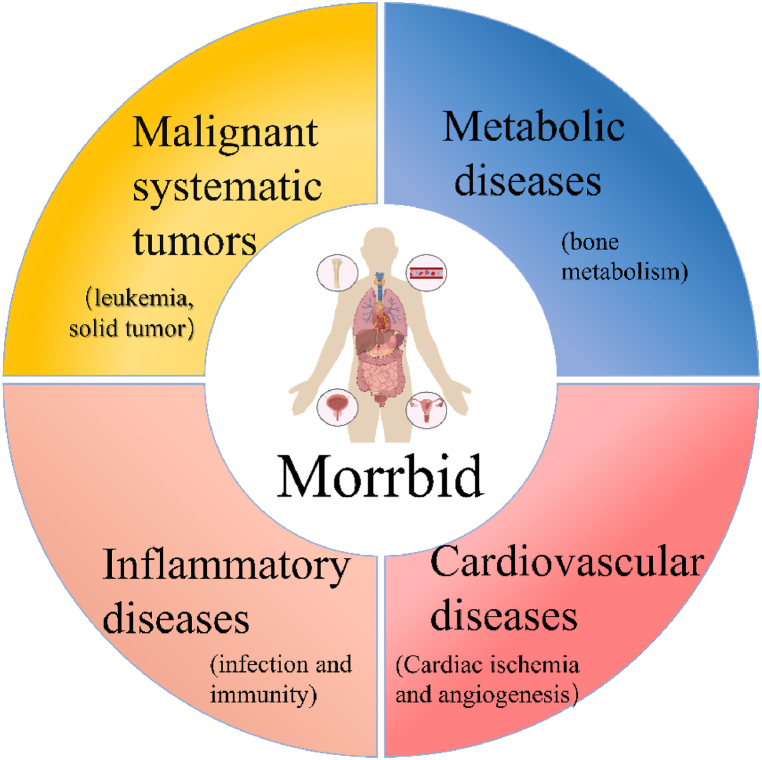
Morrbid contributes to the pathological process of multiple systemic tumors, hemooncology, inflammatory diseases, cardiovascular diseases and bone metabolism diseases.

LncRNA Morrbid is a long non-coding RNA located on the F1 arm of chromosome 2. It is conserved between species and is known as MIR4435-2 H G, AGD2, LINC00978, MIR4435-1 HG, and lncRNA-AWPPH in humans. In August 2016, Jonathan J and his colleagues published an article in Nature, revealing that Morrbid regulates the proapoptotic gene Bcl2l11 (also known as Bim) in a cis manner, extending the lifespan of short-lived bone marrow-derived innate immune cells: eosinophils, neutrophils, and Ly6C^hi^ monocytes. Consequently, it was named Myeloid RNA Regulator of Bim-Induced Death (shortened to lncRNA Morrbid) and abbreviated as Morrbid. In September of the same year, Nature Reviews Immunology published a commentary highly affirming the significance of this work. Given the revealed pathological mechanisms linking Morrbid to cancer progression, Morrbid has been regarded as an oncogene. Potential therapeutic strategies targeting Morrbid may provide novel approaches for clinicians. Therefore, it is imperative to undertake a comprehensive review of the research about Morrbid. Literature reports that lncRNA Morrbid plays a role in various tumors, cardiovascular diseases, inflammatory diseases, and bone metabolic diseases. In this work, we discuss Morrbid's involvement in each disease (Fig. 1).

## 2 Morrbid and tumors

**Fig. 2:**
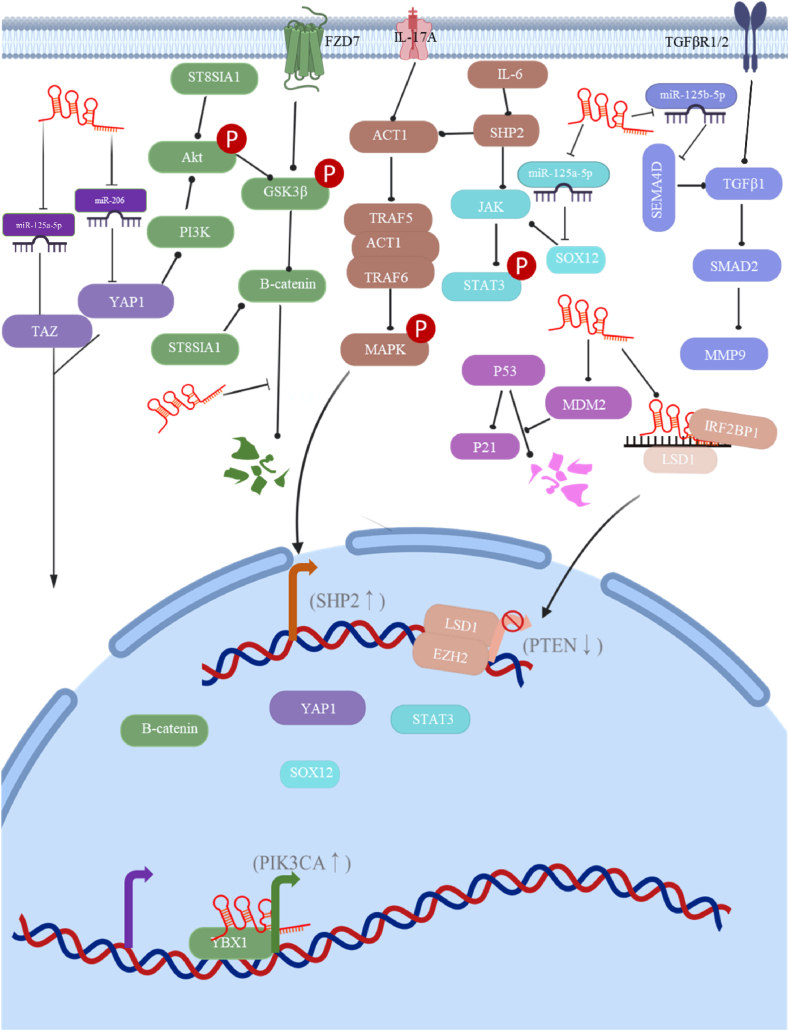
Morrbid promotes the proliferation, migration and mesenchymal transition of tumor cells through the above signaling pathways (YAP1/TAZ, PI3K/Akt, Wnt/β-catenin, MAPK/ERK, JAKs/STATs, p53 pathway and TGF-β/SMAD2 pathway.

**Fig. 3:**
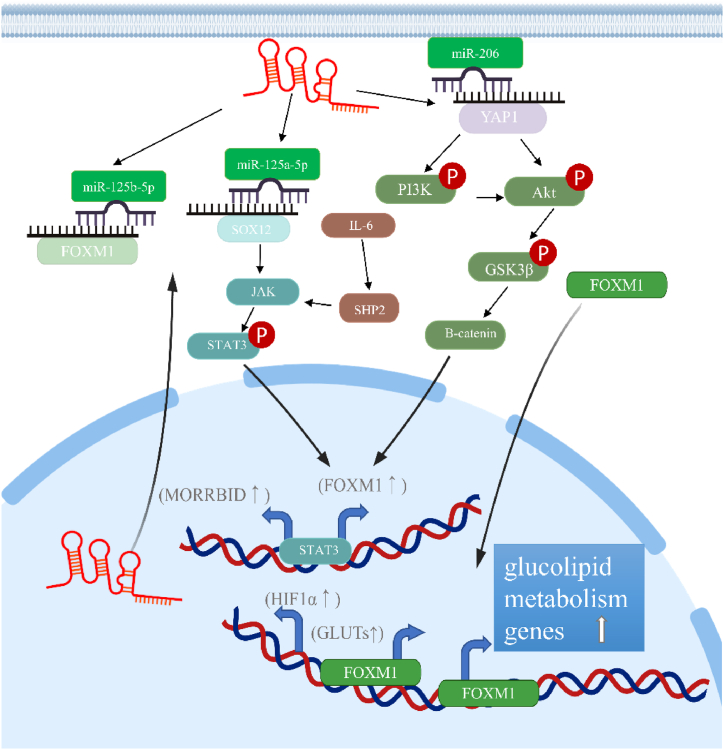
Morrbid may regulate metabolism by activating a key transcription factor FOXM1 in tumors.

Experimental studies and clinical data suggest that Morrbid plays a crucial role in the development and progression of tumors. Clinical research has reported that Morrbid is highly expressed in tumors and the serum of tumor patients. High levels of Morrbid tend to indicate more malignant tumors and are associated with TNM staging and poor prognosis. Here are two typical examples, Morrbid is higher in triple-negative breast cancer than in triple-positive breast cancer [1], and high expression of Morrbid increases the risk of death and drug resistance in children with B-cell acute Lymphoblastic Leukemia [2]. Tumor-related mechanistic studies have shown that Morrbid promotes tumorigenesis, progression, and drug resistance by altering the biological behavior of tumor cells, affecting cell survival, and altering cellular metabolism. Additionally, Morrbid can assist in tumor immune escape by inhibiting anti-tumor immunity and altering the immune microenvironment of tumors. Next, we will discuss in detail the essential contributions of Morrbid in tumorigenesis and progression from these aspects (Fig. 2, Fig. 3).

### 2.1 Morrbid regulates cancer cells' proliferation, migration, and invasion

Morrbid, like other lncRNAs, can regulate the expression of downstream genes at the DNA, mRNA, and protein levels. These processes involve altering the accessibility of specific chromatin, acting as molecular sponges, and modifying the stability of mRNAs and proteins, thereby promoting cancer cell proliferation, migration, invasion, and drug resistance. Microarray analysis showed that Morrbid is highly expressed in hepatocellular carcinoma (HCC) tissues and multiple HCC cell lines in vitro [[3], [4], [5]]. In HCC cells, Morrbid can recruit EZH2 in the nucleus, which, as a core component of polycomb repressive complex 2 (PRC2), has histone H3K27 trimethyltransferase activity and regulates the expression of genes involved in cell proliferation, cycle, and death, such as p21, Cyclin D1, and Bcl-2 [6,7]. However, other studies have found that Morrbid mainly localizes in the cytoplasm of cancer cells, such as in bladder cancer and nasopharyngeal carcinoma [[8], [9], [10], [11], [12]]. Cytoplasmic localization of Morrbid suggests its involvement in post-translational regulation of gene expression. For example, Morrbid acts as an endogenous ceRNA in the cytoplasm, competitively binding to miRNA-204 and Mir-6574–5p to promote the proliferation, migration, and invasion of non-small cell lung cancer (NSCLC) [13,14]. Moreover, Morrbid can compete with miR-22–3p and miR-21, promoting the malignant progression of breast cancer cells [15,16]. Additionally, Morrbid interacts with RNA-binding proteins (RBPs) IGF2BP1 in the cytoplasm to maintain LSD1 mRNA stability. LSD1 and EZH2 collectively bind to the PTEN promoter to induce hypermethylation at H3K27 and demethylation at H3K4. As a tumor suppressor, PTEN is repressed by the hyperactivation of Morrbid, which promotes the malignant proliferation of nasopharyngeal carcinoma cells [11]. Furthermore, Morrbid physically binds to β-catenin, protecting it from proteasomal degradation [17]. High levels of β-catenin can activate the Wnt/β-catenin signaling pathway, promoting the proliferation of NSCLC and breast cancer cells, tumor growth in vivo, and inhibiting cancer cell apoptosis [18]. In summary, Morrbid hyperactivation promotes cell survival, potentially contributing to chemoresistance, such as cisplatin resistance in NSCLC and carboplatin resistance in triple-negative breast cancer. Despite Morrbid's varying localization in different cells, its over-activation consistently acts as an oncogene. Its overall biological effect on cancer cells includes promoting proliferation, migration, survival, invasion, and drug resistance, which hampers effective cancer treatment [16,19].

### 2.2 Morrbid is involved in tumor mesenchymal transition

Epithelial-mesenchymal transition (EMT) is closely related to tumor metastasis and invasion; High EMT enhances tumor malignancy. During the process, epithelial cells transform into elongated cells with loss of adhesion molecules and acquire a mesenchymal phenotype [[20], [21], [22]]. Several studies have shown that Morrbid hyperactivation in tumors upregulates several key transcription factors of EMT and key proteins in EMT-associated signaling pathways. These transcription factors and pathway proteins form positive feedback that enhances the Morrbid-mediated effects of EMT. For example, in hepatocellular carcinoma, over-regulated Morrbid upregulates the classical EMT-associated transcription factors: Slug, Snail, and Twist1, which transcriptionally activate N-cadherin and vimentin while repressing E-cadherin expression [6,[23], [24], [25], [26]]. Additionally, Morrbid promotes vimentin translation by adsorbing miR-506–3p [27]. Morrbid adsorbing miR-506–3p also raises the content of TGFβ1 and ZEB1. TGFβ1 activates the Smad2/3 signaling pathway to upregulate vimentin expression, while ZEB1 binds to the E-box sequence in the E-cadherin promoter, leading to its downregulation, and binds to specific sequences in the vimentin promoter to activate transcription [[27], [28], [29], [30]]. Studies have shown that vimentin can feedback upregulate Snail and Slug [23,[25], [26], [27],^29^,^30^]. Furthermore, Morrbid binding with YBX1 stabilizes Snail's mRNA and binds to the promoter of PIK3CA in the nucleus to improve its transcription [3]. These mechanisms help enhance the pro-EMT effects of Morrbid in hepatocellular carcinoma. Morrbid's promotion of tumor EMT involves multiple signaling pathways [[31], [32], [33]], and the existence of signaling crossover between each pathway makes the mechanism of its promotion of EMT more complex. Lu Pei et al. confirmed that more than 200 proteins interacting with Morrbid were mainly enriched in Hippo, NF-kappa B, Wnt, mTOR, MAPK4, and JAKs pathways by RNA-pulldown experiments on T24 cells [34]. Findings from other tumors also suggest that Morrbid promotes activation of classical EMT signaling pathways such as PIK3K/AKT/mTOR, Wnt/β-catenin, JAK/STAT, TGF-β/smads, and Hippo/YAP1. For instance, Morrbid binds to the promoter of PIK3CA in the nucleus to enhance its transcription and interacts with miR-150 to upregulate GRB2-associated binder 1 (GAB1) expression, which increases the phosphorylation of ERK1/2 and AKT in hepatocellular carcinoma [3,34]. In breast and lung cancer, Morrbid upregulates β-catenin by promoting its translation and inhibiting its degradation and upregulates frizzled homolog 7 (FZD), both of which are key molecules in the Wnt signaling pathway [[35], [36], [37], [38]]. Several other studies have reported that Morrbid, by upregulating TGF-β and TGF-β receptors in tumors, activates TGF-β/smad to promote EMT [39,40]. Furthermore, Morrbid promotes EMT in rectal cancer via Hippo/YAP1 by upregulating YAP1 expression [34,41]. Studies show that down-regulation of Morrbid effectively inhibits cancer cell proliferation, survival, and EMT. For example, in gastric cancer cells, after si-Morrbid interference, the expression levels of N-Cadherin, MMP-9, VEGF, and a-SMA are significantly lower than in the sh-NC groups [18,42]. It is evident that Morrbid regulates EMT in a variety of tumors, and this regulatory effect is closely linked to its regulation of cell proliferation, migration, and invasion due to shared signaling pathways. Although the mechanism of Morrbid's pro-EMT effect is complex and requires more evidence for EMT effects in tumors, Morrbid remains a candidate target for tumor EMT based on the consistency of its EMT effects on cancer cells [18,[42], [43], [44], [45]](Fig. 2).

### 2.3 Morrbid regulates tumor immune microenvironment

Tumor microenvironment (TME), a concept for assessing tumor cells' survival environment, consists of extracellular matrix (ECM) and cell components other than cancer cells. Various types of immune cells and reactive molecules are the core of the TME and controllers during tumor development [[46], [47], [48], [49]]. Studies have shown that Morrbid hyperactivity upregulates ECM components, enhances immunosuppressive cell activity, and upregulates immune escape-associated molecules to promote tumor progression. For example, Morrbid overexpression has been shown to increase TGF-β1 and TGFβR1 levels in NSCLC and glioma, respectively. Elevated TGF-β1 and its receptor activate the TGF-β1/Smads signaling pathway, further enhancing the expression of MMP2, MMP9, and VEGF. Additionally, Morrbid directly inhibits miR-6574–5p, miR-1-3p, and miR-506–3p, thereby promoting MMP translation. MMPs, along with VEGF, contribute to tumor angiogenesis, while TGF-β attenuates antitumor immune response by inducing the differentiation of neutrophils into tumor-promoting N2 neutrophils [50,51], inhibiting CD8^+^ T cytotoxicity, and enhancing the activity of regulatory T cells (Treg) [52]. A study reported a positive correlation between Morrbid levels and the content of IL-6, implicating the IL-6/JAK2/STAT3 axis in Morrbid upregulation [53], and high-level Morrbid is associated with higher immune scores in HCC [[54], [55], [56]]. Morrbid may play an anti-tumor immune role through the IL-6/JAK2/STAT3 signaling axis. It has been reported that the IL-6/JAK2/STAT3 axis negatively regulates neutrophils, NK cells, T cells, and dendritic cells in innate immunity and promotes M1 macrophages differentiating into M2 macrophages [49,57]. M2 macrophages inactivate CD8^+^ T and NK cells through the cluster of PD-L1 [58,59], and Treg cells express cytotoxic T lymphocyte-associated protein 4 (CTLA-4), suppressing cytotoxic T cells for tumor clearance. Interestingly, Morrbid overexpression promotes M1 to M2 polarization in breast cancer, and high levels of Morrbid elevate the expression of PD-1/PD-L1/CTLA4 [[60], [61], [62]]. Additionally, cytokines such as TGF-β1 and IL-10 secreted by M2 macrophages and Treg cells aggravate the immunosuppressive effect in tumors [51,52,63]. This research uncovered that Morrbid hyperactivity mainly suppresses antitumor immunity and promotes tumor immune escape [64].

However, in contrast to most findings, Hongfei Yu et al. found that Morrbid acts as a tumor suppressor in rectal cancer [65]. They found that the knockdown of Morrbid in SW480 and RKO cells or Morrbid overexpression in DLD1 and HCT116 cells did not alter the cell biological behaviors. In vivo, they observed that the growth of MC38, a type of rectal cancer cell, was faster in Morrbid^−/−^ mice compared to WT mice. However, the growth of MC38 cells with Morrbid knockout made no difference between Morrbid^−/−^ mice and WT mice. This indicated that the microenvironment of Morrbid^−/−^ mice significantly promoted tumor growth. This conclusion was subsequently verified in a co-culture experiment of MC38 with spleen cells isolated from Morrbid^−/−^ mice and WT mice. They confirmed that the increase in polymorphonuclear myeloid-derived suppressor cells (PMN-MDSC) in the rectal tumor stroma of Morrbid^−/−^ mice might be the mechanism by which Morrbid knockout enhances antitumor immunosuppression and facilitates the progression of rectal cancer. Although this study shows that the role of Morrbid in tumor immunity is still controversial, Morrbid is indeed involved in tumor immunity.

### 2.4 Morrbid alters tumor metabolism

All the literature we consulted showed that several reports provide evidence that Morrbid is connected with tumor metabolism, especially in glucose and lipid metabolism. For instance, Morrbid silence in colon cancer results in GLUT-1 down-regulation [66]. In rectal cancer, Morrbid knockdown results in lipid accumulation in neutrophils and PMN-MDSC, which may be related to the enhanced immunosuppressive activity of PMN-MDSC [65]. These results imply that Morrbid hyperactivation may positively regulate glucose metabolism to meet the hypermetabolic characteristics of tumor growth [67]. In addition, based on the TCGA database, Morrbid, as a lncRNA related to glucose metabolism, is screened out. CeRNA network analysis reveals Morrbid hyperactivation may increase forkhead box protein M1 (FOXM1) expression by Competitively binding hsa-miR-125b-5p [68]. FOXM1 is a transcription factor (TF) of the Fox family, associated with insulin, insulin-like growth factor, leptin and adiponectin secretion, and the expression of glucose transporter type 1 (GLUT1) and glucose transporter type 2 (GLUT2) [69]. Other research groups reported that IL-6/STAT3, PI3K/Akt, Ras-ERK, YAP1/Akt/GSK3β signaling axis can activate FOXM1 expression [[70], [71], [72]], among which STAT3/FOXM1/GLUT1 signaling pathway is closely related to glycolysis [73]. Consistent with those results, Morrbid sponges miR-206 and upregulates YAP1, which can activate FOXM1 expression through the Akt/GSK3β signaling pathway in rectal cancer [41]. In short, these results indicate that Morrbid is most likely to play a crucial role in tumor glucolipid metabolism. However, the mechanism by which Morrbid regulates tumor glycolipid metabolism needs to be further elucidated (Fig. 3).

## 3 Morrbid and hematologic malignancy

Morrbid, enriched in short-lived eosinophils, neutrophils, and Ly6C^hi^ monocytes, causes H3K27me3 deposition on the Bcl2l11 promoter by working with the Polycomb repressive complex 2 (PRC2), leading to Bim downregulation [74]. These innate short-lived immune cells play a crucial role in immune response, inflammation, and repair. Leukemia is a highly malignant proliferative disease often accompanied by gene mutations, making it difficult to cure [[75], [76], [77], [78]]. Genetic susceptibility, combined with additional shocks such as infection, inflammation, radiation, and senescence, usually leads to the rapid progression of such diseases. Acute inflammatory stress itself can also cause damage or stemness loss of blood progenitors [[79], [80], [81]]. Some groups have found that Morrbid participates in hematological tumor progression.

Tet2, a member of the ten-eleven translocation dioxygenases (TETs) family, physiologically catalyzes dynamic DNA demethylation and regulates blood cell lineage determination and differentiation [82,83]. Therefore, Tet2 mutations occur in almost all hematologic tumors [84,85]. Humans with a single absence of Tet2 function may survive with a preleukemic state because Tet1/3 could partially compensate for Tet2 loss. However, LPS can induce Tet2^−/−^ mice to develop myelodysplastic syndromes (MDS) or chronic myeloid leukemia (CML) [83]. The mechanism of LPS-induced MDS or CML may be partially caused by IL-6/Shp2/Stat3 axis over-activation, leading to upregulation of Morrbid [86]. Allosteric inhibition of Shp2 (SHP099, a pan-Stat3 inhibitor) or Morrbid knockout can alleviate abnormal clonal hematopoiesis in Tet2^−/−^ mice treated with LPS [53]. Additionally, juvenile myelomonocytic leukemia (JMML) is a hyperinflammatory syndrome, and allogeneic hematopoietic stem cell transplantation is the only effective treatment. 35 % of JMML cases are caused by PTPN11 encoding SHP2 mutation. According to a study, Shp2 and Morrbid biallelic knockout can alleviate anemia and reduce inflammatory cells in the bone marrow of mice, with a longer median survival compared to single Shp2 knockout mice [87]. A high level of Morrbid in human JMML with PTPN11 mutations reflects a lower overall survival rate [87].

Two other studies focus on the Flt3 mutation associated with AML, which encodes the type III receptor tyrosine kinase [88,89]. Flt3-ITD refers to different lengths of tandem duplications inserted into the Flt3 near the membrane structure domain [90,91]. FLT3-ITD activates multiple signaling pathways downstream of FLT3, such as RAS/MAPK, PI3K/AKT, and JAK/STAT, leading to blood cell proliferation, survival, and inhibition of apoptosis [88,89,92]. Zhigang Cai and his colleagues found that Morrbid hyperactivation occurs in Tet2^−/−^; Flt3-ITD mice. Morrbid knockout significantly reduced the percentage of leukemia cells in the bone marrow and peripheral blood and alleviated extramedullary infiltration in mice. Additionally, Morrbid interacts with Rho-associated protein kinase 2 (ROCK2) to promote abnormal T lymphocyte proliferation and inhibit its apoptosis [93]. These studies indicate that Morrbid may be a new target for hematological malignancy treatment in synergy with other drugs [78].

## 4 Morrbid and inflammatory diseases

Based on the findings of overexpression of Morrbid in various tumors, it has been shown that aberrant activation of Morrbid is closely related to immune cells and inflammation. The IL-6/Shp2/Stat3 axis upregulates Morrbid, which subsequently downregulates Bim to prolong the lifespan of intrinsic immune cells [74], favoring the expansion of the immune range and enhancement of immune competence [94,95]. This observation suggests that Morrbid regulation of immunity is a double-edged sword in different disease states. For example, the increased immune activity of dendritic cells (DCs) in individuals with spontaneous highly antiretroviral replication may be due to the upregulation of mTORC1 by Morrbid hyperactivation through H3K27ac enrichment at the RPTOR promoter, resulting in enhanced beta-oxidation and oxidative phosphorylation [96]. Morrbid knockout causes mice to be highly susceptible to L. monocytogenes infection [74]. Additionally, acute and chronic lymphocytic choriomeningitis virus (LCMV) infection induces CD8^+^ T cell activation and significant upregulation of Morrbid. Morrbid knockdown results in a significantly higher number of CD8^+^ T cells in the peripheral circulation compared to wild-type (WT) mice after LCMV infection. The proportions of IFN-γ, TNF-α, and granzyme B (GzmB) T cells, as well as the subpopulation of memory T cells, are significantly increased [97]. This suggests that Morrbid restricts CD8^+^ T cell numbers under physiological conditions and is associated with maintaining CD8^+^ T cell function homeostasis.

Abnormally upregulated Morrbid activates the NF-κB signaling pathway through RNA-binding protein heterogeneous nuclear ribonucleoprotein A1 (HNRNPA1) [98], which is involved in angiogenesis and inflammatory response. Additionally, Morrbid may be involved in the pathological processes of autoimmune diseases such as recurrent periodontitis, autoimmune encephalomyelitis, and ulcerative colitis [[99], [100], [101], [102], [103]].

In conclusion, Morrbid is required to maintain an appropriate inflammatory and cellular immune response. Dysregulation of Morrbid expression may be one of the pathological mechanisms involved in the development and progression of autoimmune diseases, but the molecular mechanisms of Morrbid involvement in autoimmune diseases need to be further elucidated.

## 5 Morrbid and cardiovascular diseases

Cardiovascular diseases (CVD) have become the leading cause of death worldwide [104,105]. As early as 2016, Morrbid was identified as a lncRNA associated with myocardial hypertrophy [106]. Although the mechanism by which Morrbid participates in cardiac hypertrophy remains unclear, studies have reported that Morrbid is significantly downregulated in transverse aortic constriction (TAC) mice with adenosine receptor A2A overexpression, suggesting that Morrbid may mediate the cardioprotective effect of A2A or directly participate in the pathological process of cardiac hypertrophy [106]. Clinical data also show that Morrbid levels rise significantly in the peripheral blood of patients with coronary artery diseases and in the carotid atherosclerotic plaques of patients with type 2 diabetes mellitus. Multivariate logistic regression analysis indicates that Morrbid level in serum is an independent risk factor for coronary artery disease diagnosis and prognosis, and statins can reduce Morrbid levels in serum [107]. A recent study has reported that Morrbid acts as a modulator of monocyte-macrophage phenotypes, which are involved in atherogenesis, suggesting that Morrbid is a potential diagnostic and prognostic index for atherosclerosis [108]. This suggesting that Morrbid is a potential diagnostic and prognostic index for atherosclerosis.

Additionally, Morrbid has been found to be involved in the pathology of ischemic heart injury. In mice with 24-h infarction, Morrbid was significantly upregulated and exerted a protective effect on the heart through Serpine1 [109], a molecule associated with blood vessels and coagulation. The cardioprotective effect of Morrbid targeting Serpine1 may be related to improved microcirculation. Another study showed that Morrbid was upregulated in the plasma of reperfused patients and in the cardiac tissues of I/R (30 min of ischemia followed by 2 h of reperfusion) mice. In this context, upregulated Morrbid targeted mitochondrial fission protein 1 (MTFP1) in cardiomyocytes via MiR-125a-5p, promoting cardiac injury during I/R [[109], [110], [111], [112], [113], [114], [115], [116], [117], [118]]. These results suggest that Morrbid may target different molecules with different functions in various heart disease models.

## 6 Morrbid and metabolic diseases

As stated earlier, Morrbid regulates glucolipid metabolism in tumor cells and immune cells, so redundant details will be omitted here. This section introduces Morrbid's role in bone metabolic disease pathology. Several pieces of evidence have shown that Morrbid is necessary for physiological bone metabolism, and its down-regulation promotes the development of bone metabolic diseases [119,120]. Morrbid down-regulation in the serum of osteoporosis patients leads to an imbalance in the ratio of type I collagen a1 and α2 in osteoblasts and bone microstructural abnormalities [121,122]. BMP-2, an osteoblast differentiation inducer, induces Morrbid and runt-related transcription factor 2 (RUNX2) expression in human bone marrow mesenchymal stem cells (hMSC-BM). RUNX2, a fundamental transcription factor for bone metabolism and development, works with Morrbid to promote chondrocyte proliferation and anti-apoptotic effects [123]. Runx2 itself promotes chondrocyte differentiation into osteoblasts [123,124]. RUNX2 itself promotes the differentiation of chondrocytes into osteoblasts. Additionally, Morrbid overexpression promotes chondrocyte survival in osteoarthritis by targeting miR-510–3p/IL-17A expression [125]. In summary, these findings indicate that Morrbid reduction is harmful to the maintenance of bone microstructure, while Morrbid overexpression promotes chondrogenic differentiation and osteogenesis.

## 7 Discussion

As early as 2015, Morrbid was reported to be involved in the pathological processes of lung cancer and cardiac hypertrophy [106,126]. Morrbid became famous in 2016 for its involvement in regulating the life span of circulating short-lived cells [127]. Subsequently, numerous studies have been conducted on various tumors, cardiovascular diseases, inflammatory diseases, and bone metabolism.

Oncological research has revealed that Morrbid is over-activated in human tumor tissues, with its serum levels positively correlated with tumor malignancy and poor prognosis. Mechanistic studies have shown that Morrbid overexpression promotes cancer cell proliferation, migration, epithelial-mesenchymal transition (EMT), immune escape, and drug resistance [43,128,129]. Thus, Morrbid serum levels could serve as an indicator for clinical cancer diagnosis and prognosis, and targeting Morrbid or using it in combination with antitumor therapies offers new insights for clinicians.

Furthermore, studies on hematologic neoplasms and inflammatory diseases have shown that physiological levels of Morrbid are essential for a normal immune response and for limiting excessive inflammatory responses. Therefore, one of the challenges in targeting Morrbid for antineoplastic drug therapy is the potential risk of inducing excessive inflammatory responses or immunosuppression. Additionally, Morrbid serum levels may also serve as indicators for the development and progression of atherosclerosis-related diseases and the efficacy of statin therapy.

However, in a study on rectal cancer by Hongfei Yu and colleagues, it was found that Morrbid was localized in the mesenchyme of rectal cancer rather than in the cancer cells themselves [65]. Knocking down or overexpressing Morrbid in rectal cancer cells did not affect their biological behavior. Contrary to most tumor studies, this study suggests that Morrbid exerts an inhibitory effect, and Morrbid deficiency actually promotes tumor progression [65]. The authors discussed that the inconsistent results might be due to other studies not accounting for lncRNA CYTOR, a highly homologous lncRNA to human Morrbid, which is also highly expressed in tumor tissues and promotes tumor progression [65,[130], [131], [132], [133], [134], [135]]. Searches in the NCBI and Ensembl databases confirmed sequence homology between human lncRNA CYTOR and Morrbid. The NCBI database even lists Cytor as one of the Morrbid aliases in mice, suggesting shared functions between the two. Therefore, species and primer specificity should be considered when designing primers for Morrbid. However, there are controversial points in Yu's study [65]. For instance, the expected efficiency of RNAi interference with low Morrbid expression in rectal cancer cells may not have been achieved. Additionally, the overexpression levels of Morrbid might have surpassed physiological conditions, potentially activating certain signaling pathways and altering cellular behavior. Even if Morrbid is localized in the interstitial cells of cancer tissue, it could affect rectal cancer cells through intercellular communication, such as via exosomes.

In summary, most Morrbid research has focused on tumors. However, it is evident that more work is needed to fully understand Morrbid's role in metabolism regulation, inflammation modulation, immune regulation, and involvement in autoimmune diseases. This underscores the urgency and importance of further research in these areas and the potential for significant contributions to our understanding of Morrbid's functions and its implications for disease.

## CRediT authorship contribution statement

**Haiqiong Yang:** Writing – original draft. **Jiali Gao:** Data curation. **Zaiyong Zheng:** Writing – review & editing. **Yang Yu:** Writing – review & editing. **Chunxiang Zhang:** Supervision, Project administration, Funding acquisition.

## Declaration of competing interest

The authors declare the following financial interests/personal relationships which may be considered as potential competing interests:Chunxiang Zhang reports financial support was provided by 10.13039/501100001809National Natural Science Foundation of China, China. If there are other authors, they declare that they have no known competing financial interests or personal relationships that could have appeared to influence the work reported in this paper.

## References

[1] Marziyeh Bayat H.N., Mohammad Fazilati, Hossein Hejazi, Mohammd Ghaedi MaK. (2023). Association of MIR4435-2HG expression with TP53 mutation, estrogen receptor activity, and poor prognosis in breast cancer. Jentashapir J. Cell Mol. Biol..

[2] Torres-Llanos Y., Zabaleta J., Cruz-Rodriguez N. (2024). As a possible novel predictive biomarker of chemotherapy response and death in pediatric B-cell ALL. Front. Mol. Biosci..

[3] Zhao X.D., Liu Y.B., Yu S. (2017). Long noncoding RNA AWPPH promotes hepatocellular carcinoma progression through YBX1 and serves as a prognostic biomarker. Biochim. Biophys. Acta, Mol. Basis Dis..

[4] Kong Q.L., Liang C.Q., Jin Y. (2019). The lncRNA MIR4435-2HG is upregulated in hepatocellular carcinoma and promotes cancer cell proliferation by upregulating miRNA-487a. Cell. Mol. Biol. Lett..

[5] Wang B., Tang D.Y., Zhang Z.Y. (2020). Identification of aberrantly expressed lncRNA and the associated TF-mRNA network in hepatocellular carcinoma. J. Cell. Biochem..

[6] Xu X.Y., Gu J.M., Ding X.G. (2019). LINC00978 promotes the progression of hepatocellular carcinoma by regulating EZH2-mediated silencing of p21 and E-cadherin expression. Cell Death Dis..

[7] Anzalone G., Moscato M., Montalbano A.M. (2021). PBDEs affect inflammatory and oncosuppressive mechanisms via the EZH2 methyltransferase in airway epithelial cells. Life Sci..

[8] Wang W., Xu Z.H., Wang J.Y. (2019). LINC00978 promotes bladder cancer cell proliferation, migration and invasion by sponging miR-4288. Mol. Med. Rep..

[9] Hu Z.Q., Ma S.Q., Sun Y. (2022). Identification of long non-coding RNA mir4435-2HG as a prognostic biomarker in bladder cancer. Genes.

[10] Huang G.M., Huang Y.W., Zhang C.Y. (2022). Identification of cuproptosis-related long noncoding RNA signature for predicting prognosis and immunotherapy response in bladder cancer. Sci. Rep..

[11] Guo D.Q., Liu F., Zhang L. (2021). Long non-coding RNA AWPPH enhances malignant phenotypes in nasopharyngeal carcinoma via silencing PTEN through interacting with LSD1 and EZH2. Biochem. Cell. Biol..

[12] Chen W., Du M.Y., Hu X.Y. (2020). Long noncoding RNA cytoskeleton regulator RNA promotes cell invasion and metastasis by titrating miR-613 to regulate ANXA2 in nasopharyngeal carcinoma. Cancer Med..

[13] Li X.L., Ren Y., Zuo T. (2018). Long noncoding RNA LINC00978 promotes cell proliferation and invasion in non-small cell lung cancer by inhibiting miR-6754-5p. Mol. Med. Rep..

[14] Wu D., Qin B.Y., Qi X.G. (2020). LncRNA AWPPH accelerates the progression of non-small cell lung cancer by sponging miRNA-204 to upregulate CDK6. Eur. Rev. Med. Pharmacol. Sci..

[15] Ke J., Wang Q.H., Zhang W. (2022). LncRNA MIR4435-2HG promotes proliferation, migration, invasion and epithelial mesenchymal transition via targeting miR-22-3p/TMEM9B in breast cancer. Am. J. Trans. Res..

[16] Liu A.N., Qu H.J., Gong W.J. (2019). LncRNA AWPPH and miRNA-21 regulates cancer cell proliferation and chemosensitivity in triple-negative breast cancer by interacting with each other. J. Cell. Biochem..

[17] Qian H.Y., Chen L., Huang J.P. (2018). The lncRNA MIR4435-2HG promotes lung cancer progression by activating β-catenin signalling. J. Mol. Med..

[18] Chen D.Q., Tang P.T., Wang Y.K. (2021). Downregulation of long non-coding RNA MR4435-2HG suppresses breast cancer progression via the Wnt/β-catenin signaling pathway. Oncol. Lett..

[19] Luo P., Wu S.G., Ji K.B. (2020). LncRNA MIR4435-2HG mediates cisplatin resistance in HCT116 cells by regulating Nrf2 and HO-1. PLoS One.

[20] Gurzu S., Silveanu C., Fetyko A. (2016). Systematic review of the old and new concepts in the epithelial-mesenchymal transition of colorectal cancer. World J. Gastroenterol..

[21] Nieto M.A., Huang R.Y., Jackson R.A. (2016). Emt: 2016. Cell.

[22] Bakir B., Chiarella A.M., Pitarresi J.R. (2020). EMT, MET, plasticity, and tumor metastasis. Trends Cell Biol..

[23] Scanlon C.S., Van Tubergen E.A., Inglehart R.C. (2013). Biomarkers of epithelial-mesenchymal transition in squamous cell carcinoma. J. Dent. Res..

[24] Georgakopoulos-Soares I., Chartoumpekis D.V., Kyriazopoulou V. (2020). EMT factors and metabolic pathways in cancer. Front. Oncol..

[25] Strouhalova K., Prechova M., Gandalovicova A. (2020). Vimentin intermediate filaments as potential target for cancer treatment. Cancers.

[26] Usman S., Waseem N.H., Nguyen T.K.N. (2021). Vimentin is at the heart of epithelial mesenchymal transition (EMT) mediated metastasis. Cancers.

[27] Li S.L., Hu X.W., Yu S.M. (2023). Hepatic stellate cell-released CXCL1 aggravates HCC malignant behaviors through the MIR4435-2HG/miR-506-3p/TGFB1 axis. Cancer Sci..

[28] Zhu W.W., Wang J., Liu X. (2022). lncRNA CYTOR promotes aberrant glycolysis and mitochondrial respiration via HNRNPC-mediated ZEB1 stabilization in oral squamous cell carcinoma. Cell Death Dis..

[29] Zhang P., Sun Y., Ma L. (2015). ZEB1: at the crossroads of epithelial-mesenchymal transition, metastasis and therapy resistance. Cell Cycle.

[30] Qin Y., Yu J., Zhang M. (2019). ZEB1 promotes tumorigenesis and metastasis in hepatocellular carcinoma by regulating the expression of vimentin. Mol. Med. Rep..

[31] Chen Z., Guan D., Zhu Q. (2023). Biological roles and pathogenic mechanisms of LncRNA mir4435-2HG in cancer: a comprehensive review. Curr. Issues Mol. Biol..

[32] Li S., Yao W.P., Liu R.Q. (2022). Long non-coding RNA LINC00152 in cancer: roles, mechanisms, and chemotherapy and radiotherapy resistance. Front. Oncol..

[33] Qian P., Xu Z., Chen H. (2020). Abnormally expressed lncRNAs in the prognosis and clinicopathology of oesophageal cancer: a systematic review and meta-analysis. J. Genet..

[34] Pei L., Yan D., He Q.Q. (2023). LncRNA MIR4435-2HG drives cancer progression by modulating cell cycle regulators and mTOR signaling in stroma-enriched subtypes of urothelial carcinoma of the bladder. Cell. Oncol..

[35] Li C., Wang F., Wei B. (2019). LncRNA AWPPH promotes osteosarcoma progression via activation of Wnt/β-catenin pathway through modulating miR-93-3p/FZD7 axis. Biochem. Biophys. Res. Commun..

[36] Song Z., Du J.F., Zhou L.R. (2019). lncRNA AWPPH promotes proliferation and inhibits apoptosis of non-small cell lung cancer cells by activating the Wnt/-catenin signaling pathway. Mol. Med. Rep..

[37] Wang H.Y., Wu M.J., Lu Y.M. (2019). LncRNA MIR4435-2HG targets desmoplakin and promotes growth and metastasis of gastric cancer by activating Wnt/β-catenin signaling. Aging-US.

[38] Yu G.Y., Wang W.S., Deng J.F. (2019). LncRNA AWPPH promotes the proliferation, migration and invasion of ovarian carcinoma cells via activation of the Wnt/-catenin signaling pathway. Mol. Med. Rep..

[39] Dai B., Xiao Z.Y., Mao B.B. (2019). lncRNA AWPPH promotes the migration and invasion of glioma cells by activating the TGF-β pathway. Oncol. Lett..

[40] Fu M., Huang Z.H., Zang X.Y. (2018). Long noncoding RNA LINC00978 promotes cancer growth and acts as a diagnostic biomarker in gastric cancer. Cell Prolif..

[41] Dong X.H., Yang Z., Yang H.W. (2020). Long non-coding RNA mir4435-2HG promotes colorectal cancer proliferation and metastasis through miR-206/YAP1 Axis. Front. Oncol..

[42] Ghafouri-Fard S., Askari A., Hussen B.M. (2023). A review on the role of LINC00152 in different disorders. Pathol. Res. Pract..

[43] Zhong C.M., Xie Z.J., Zeng L.H. (2022). MIR4435-2HG is a potential pan-cancer biomarker for diagnosis and prognosis. Front. Immunol..

[44] Ghasemian M., Rajabibazl M., Sahebi U. (2022). Long non-coding RNA MIR4435-2HG: a key molecule in progression of cancer and non-cancerous disorders. Cancer Cell Int..

[45] Ghasemian M., Rajabibazl M., Mirfakhraie R. (2021). Long noncoding RNA LINC00978 acts as a potential diagnostic biomarker in patients with colorectal cancer. Exp. Mol. Pathol..

[46] Balkwill F.R., Capasso M., Hagemann T. (2012). The tumor microenvironment at a glance. J. Cell Sci..

[47] Baghban R., Roshangar L., Jahanban-Esfahlan R. (2020). Tumor microenvironment complexity and therapeutic implications at a glance. Cell Commun. Signal..

[48] Anderson N.M., Simon M.C. (2020). The tumor microenvironment. Curr. Biol..

[49] Yenyuwadee S., Aliazis K., Wang Q. (2022). Immune cellular components and signaling pathways in the tumor microenvironment. Semin. Cancer Biol..

[50] Masucci M.T., Minopoli M., Carriero M.V. (2019). Tumor associated neutrophils. Their role in tumorigenesis, metastasis, prognosis and therapy. Front. Oncol..

[51] Nagarsheth N., Wicha M.S., Zou W. (2017). Chemokines in the cancer microenvironment and their relevance in cancer immunotherapy. Nat. Rev. Immunol..

[52] Maimela N.R., Liu S., Zhang Y. (2019). Fates of CD8+ T cells in tumor microenvironment. Comput. Struct. Biotechnol. J..

[53] Cai Z.G., Kotzin J., Ramdas B. (2018). Downregulation of Morrbid in tet2-deficient preleukemic cells overcomes resistance to inflammatory stress and mitigates clonal hematopoiesis. Blood.

[54] Bai Y., Lin H.P., Chen J.Q. (2021). Identification of prognostic glycolysis-related lncRNA signature in tumor immune microenvironment of hepatocellular carcinoma. Front. Mol. Biosci..

[55] Mao G.C., Li L., Shan C.Y. (2022). High expression of RRM2 mediated by non-coding RNAs correlates with poor prognosis and tumor immune infiltration of hepatocellular carcinoma. Front. Med..

[56] Mao G.C., Shan C.Y., Li W.M. (2022). High expression of RRM1 mediated by ncRNAs correlates with poor prognosis and tumor immune infiltration of hepatocellular carcinoma. Int. J. Gen. Med..

[57] Del Prete A., Salvi V., Soriani A. (2023). Dendritic cell subsets in cancer immunity and tumor antigen sensing. Cell. Mol. Immunol..

[58] Boutilier A.J., Elsawa S.F. (2021). Macrophage polarization states in the tumor microenvironment. Int. J. Mol. Sci..

[59] Abaza A., Sid Idris F., Anis Shaikh H. (2023). Programmed cell death protein 1 (PD-1) and programmed cell death ligand 1 (PD-L1) immunotherapy: a promising breakthrough in cancer therapeutics. Cureus.

[60] Lv W.C., Tan Y.F., Zhou X.M. (2022). Landscape of prognosis and immunotherapy responsiveness under tumor glycosylation-related lncRNA patterns in breast cancer. Front. Immunol..

[61] Jiang H.W., Sun J.X., Liu F.C. (2022). An immune-related long noncoding RNA pair as a new biomarker to predict the prognosis of patients in breast cancer. Front. Genet..

[62] Li C.F., Chen Z.J., Gao J.L. (2022). MIR4435-2HG in exosomes promotes gastric carcinogenesis by inducing M2 polarization in macrophages. Front. Oncol..

[63] Kim H.R., Park H.J., Son J. (2019). Tumor microenvironment dictates regulatory T cell phenotype: upregulated immune checkpoints reinforce suppressive function. J. Immunother. Cancer.

[64] Quail D.F., Joyce J.A. (2013). Microenvironmental regulation of tumor progression and metastasis. Nat. Med..

[65] Yu H.F., Chen C.Y., Han F.Y. (2022). Long noncoding RNA mir4435-2HG suppresses colorectal cancer initiation and progression by reprogramming neutrophils. Cancer Immunol. Res..

[66] Bai J., Xu J., Zhao J. (2019). Downregulation of lncRNA AWPPH inhibits colon cancer cell proliferation by downregulating GLUT-1. Oncol. Lett..

[67] Wei Z., Xia J., Li J. (2023). SIRT1 promotes glucolipid metabolic conversion to facilitate tumor development in colorectal carcinoma. Int. J. Biol. Sci..

[68] Xu Z.W., Pei C.Z., Cheng H.J. (2023). Comprehensive analysis of FOXM1 immune infiltrates, m6a, glycolysis and ceRNA network in human hepatocellular carcinoma. Front. Immunol..

[69] Zhang H., Ackermann A.M., Gusarova G.A. (2006). The FoxM1 transcription factor is required to maintain pancreatic beta-cell mass. Mol. Endocrinol..

[70] Mahmoodzadeh Sagheb M., Azarpira N., Mokhtary M. (2013). The effects of leptin and adiponectin on Pdx1, Foxm1, and PPARgamma transcription in rat islets of langerhans. Hepat. Mon..

[71] Saavedra-Garcia P., Nichols K., Mahmud Z. (2018). Unravelling the role of fatty acid metabolism in cancer through the FOXO3-FOXM1 axis. Mol. Cell. Endocrinol..

[72] Shang R., Wang M., Dai B. (2020). Long noncoding RNA SLC2A1-AS1 regulates aerobic glycolysis and progression in hepatocellular carcinoma via inhibiting the STAT3/FOXM1/GLUT1 pathway. Mol. Oncol..

[73] Zhao B., Li M., Su Y. (2023). Role of transcription factor FOXM1 in diabetes and its complications. Int. J. Mol. Med..

[74] Kotzin J.J., Spencer S.P., Mccright S.J. (2016). The long non-coding RNA Morrbid regulates Bim and short-lived myeloid cell lifespan. Nature.

[75] Lang T.J.L., Damm F., Bullinger L. (2023). Mechanisms of resistance to small molecules in acute myeloid leukemia. Cancers.

[76] Perl A.E., Altman J.K., Cortes J. (2017). Selective inhibition of FLT3 by gilteritinib in relapsed or refractory acute myeloid leukaemia: a multicentre, first-in-human, open-label, phase 1-2 study. Lancet Oncol..

[77] Nuno K.A., Azizi A., Kohnke T. (2023). Convergent epigenetic evolution drives relapse in acute myeloid leukemia. bioRxiv.

[78] Tang L., Huang Z., Mei H. (2023). Immunotherapy in hematologic malignancies: achievements, challenges and future prospects. Signal Transduct. Targeted Ther..

[79] Abkowitz J.L. (2014). Clone wars--the emergence of neoplastic blood-cell clones with aging. N. Engl. J. Med..

[80] Kobayashi H., Suda T., Takubo K. (2016). How hematopoietic stem/progenitors and their niche sense and respond to infectious stress. Exp. Hematol..

[81] Zhao J.L., Baltimore D. (2015). Regulation of stress-induced hematopoiesis. Curr. Opin. Hematol..

[82] Ko M., Bandukwala H.S., An J. (2011). Ten-Eleven-Translocation 2 (TET2) negatively regulates homeostasis and differentiation of hematopoietic stem cells in mice. Proc. Natl. Acad. Sci. U. S. A..

[83] Lazarenkov A., Sardina J.L. (2022). Dissecting TET2 regulatory networks in blood differentiation and cancer. Cancers.

[84] Abegunde S.O., Buckstein R., Wells R.A. (2018). An inflammatory environment containing TNFalpha favors Tet2-mutant clonal hematopoiesis. Exp. Hematol..

[85] Baessler A., Novis C.L., Shen Z. (2022). Tet2 coordinates with Foxo1 and Runx1 to balance T follicular helper cell and T helper 1 cell differentiation. Sci. Adv..

[86] Cai Z.G., Kotzin J.J., Ramdas B. (2018). Inhibition of inflammatory signaling in Tet2 mutant preleukemic cells mitigates stress-induced abnormalities and clonal hematopoiesis. Cell Stem Cell.

[87] Cai Z.G., Zhang C., Kotzin J.J. (2020). Role of lncRNA Morrbid in PTPN11(Shp2)E76K-driven juvenile myelomonocytic leukemia. Blood Adv..

[88] Zhao J.C., Agarwal S., Ahmad H. (2022). A review of FLT3 inhibitors in acute myeloid leukemia. Blood Rev..

[89] Jalte M., Abbassi M., El Mouhi H. (2023). FLT3 mutations in acute myeloid leukemia: unraveling the molecular mechanisms and implications for targeted therapies. Cureus.

[90] Roskoski R. (2020). The role of small molecule Flt3 receptor protein-tyrosine kinase inhibitors in the treatment of Flt3-positive acute myelogenous leukemias. Pharmacol. Res..

[91] Alarbeed I.F., Wafa A., Moassass F. (2021). De novo adult acute myeloid leukemia with two new mutations in juxtatransmembrane domain of the FLT3 gene: a case report. J. Med. Case Rep..

[92] Kennedy V.E., Smith C.C. (2020). FLT3 mutations in acute myeloid leukemia: key concepts and emerging controversies. Front. Oncol..

[93] Li X.H., Song F.F., Sun H.Q. (2020). Long non-coding RNA AWPPH interacts with ROCK2 and regulates the proliferation and apoptosis of cancer cells in pediatric T-cell acute lymphoblastic leukemia. Oncol. Lett..

[94] Zhao Y., Zou W., Du J. (2018). The origins and homeostasis of monocytes and tissue-resident macrophages in physiological situation. J. Cell. Physiol..

[95] Qu L., Matz A.J., Karlinsey K. (2022). Macrophages at the crossroad of meta-inflammation and inflammaging. Genes.

[96] Hartana C.A., Rassadkina Y., Gao C. (2021). Long noncoding RNA MIR4435-2HG enhances metabolic function of myeloid dendritic cells from HIV-1 elite controllers. J. Clin. Invest..

[97] Kotzin J.J., Iseka F., Wright J. (2019). The long noncoding RNA Morrbid regulates CD8 T cells in response to viral infection. Proc. Natl. Acad. Sci. U. S. A..

[98] Li Z., Cao Z., Li N. (2023). M2 macrophage-derived exosomal lncRNA mir4435-2HG promotes progression of infantile hemangiomas by targeting HNRNPA1. Int. J. Nanomed..

[99] Cao L., Tan Q.H., Zhu R. (2023). LncRNA MIR4435-2HG suppression regulates macrophage M1/M2 polarization and reduces intestinal inflammation in mice with ulcerative colitis. Cytokine.

[100] Lu J.W., Rouzigu A., Teng L.H. (2021). The construction and comprehensive analysis of inflammation-related ceRNA networks and tissue-infiltrating immune cells in ulcerative progression. BioMed Res. Int..

[101] Fenton C.G., Ray M.K., Meng W. (2023). Methylation-regulated long non-coding RNA expression in ulcerative colitis. Int. J. Mol. Sci..

[102] Wang X.F., Ma F., Jia P.Z. (2019). LncRNA AWPPH overexpression predicts the recurrence of periodontitis. Biosci. Rep..

[103] Zohar K., Lezmi E., Reichert F. (2023). Coordinated transcriptional waves define the inflammatory response of primary microglial culture. Int. J. Mol. Sci..

[104] Poopak A., Saeedi Moghaddam S., Esfahani Z. (2023). National and subnational burden of leukemia and its risk factors, 1990-2019: results from the Global Burden of Disease study 2019. PLoS One.

[105] Wen F., Jiang S., Yuan P. (2023). Vascular health promotion Project and vascular medicine in China-CCVM2004-2023. Vasc. Health Risk Manag..

[106] Zhang L., Hamad E.A., Vausort M. (2015). Identification of candidate long noncoding RNAs associated with left ventricular hypertrophy. CTS-Clin. Transl. Sci..

[107] Yu T., Xu B.F., Bao M.H. (2022). Identification of potential biomarkers and pathways associated with carotid atherosclerotic plaques in type 2 diabetes mellitus: a transcriptomics study. Front. Endocrinol..

[108] Xiang D., Jiang L., Yuan Q. (2023). Leukocyte-specific Morrbid promotes leukocyte differentiation and atherogenesis. Research.

[109] Yu Y., Yang H.Q., Li Q.T. (2023). Stress-enhanced cardiac lncRNA Morrbid protects hearts from acute myocardial infarction. JCI Insight.

[110] Wang X.L., Ren L.N., Chen S. (2022). Long non-coding RNA MIR4435-2HG/microRNA-125a-5p axis is involved in myocardial ischemic injuries. Bioengineered.

[111] Panigrahi D.P., Praharaj P.P., Behera B.P. (2023). The inner mitochondrial membrane fission protein MTP18 serves as a mitophagy receptor to prevent apoptosis in oral cancer. J. Cell Sci..

[112] Pan S., Zhou J., Yang W. (2023). MiR-125b-5p targets MTFP1 to inhibit cell proliferation, migration, and invasion and facilitate cell apoptosis in endometrial carcinoma. Mol. Biotechnol..

[113] Donnarumma E., Kohlhaas M., Vimont E. (2022). Mitochondrial Fission Process 1 controls inner membrane integrity and protects against heart failure. Nat. Commun..

[114] Xiao T., Sun J., Xing Z. (2020). MTFP1 overexpression promotes the growth of oral squamous cell carcinoma by inducing ROS production. Cell Biol. Int..

[115] Siebert A.E., Brake M.A., Verbeek S.C. (2023). Identification of genomic loci regulating platelet plasminogen activator inhibitor-1 in mice. J. Thromb. Haemostasis.

[116] Eren M., Boe A.E., Klyachko E.A. (2014). Role of plasminogen activator inhibitor-1 in senescence and aging. Semin. Thromb. Hemost..

[117] Van De Craen B., Declerck P.J., Gils A. (2012). The Biochemistry, Physiology and Pathological roles of PAI-1 and the requirements for PAI-1 inhibition in vivo. Thromb. Res..

[118] He W., Gu L., Yang J. (2023). Exosomal circCNOT6L regulates astrocyte apoptotic signals induced by hypoxia exposure through miR99a-5p/SERPINE1 and alleviates ischemic stroke injury. Mol. Neurobiol..

[119] Patel H., Shrivastava S. (2023). Osteogenesis imperfecta: an unusual presentation. Pan Afr. Med. J..

[120] Zhang L., Guan Q., Wang Z. (2023). Consequences of aging on bone. Aging Dis. 15.

[121] Qian G., Yu Y.M., Dong Y.H. (2022). LncRNA AWPPH is downregulated in osteoporosis and regulates type I collagen α1 and α2 ratio. Arch. Physiol. Biochem..

[122] Xiao Y., Bao Y.C., Tang L. (2019). LncRNA MIR4435-2HG is downregulated in osteoarthritis and regulates chondrocyte cell proliferation and apoptosis. J. Orthop. Surg. Res..

[123] Chen X.T., Li J., Liang D.W. (2020). LncRNA AWPPH participates in the development of non-traumatic osteonecrosis of femoral head by upregulating Runx2. Exp. Ther. Med..

[124] Xiao Y., Xie X., Chen Z. (2023). Advances in the roles of ATF4 in osteoporosis. Biomed. Pharmacother..

[125] Liu Y.L., Yang Y., Ding L.J. (2020). LncRNA MIR4435-2HG inhibits the progression of osteoarthritis through miR-510-3p sponging. Exp. Ther. Med..

[126] Yang Q., Xu E., Dai J. (2015). A novel long noncoding RNA AK001796 acts as an oncogene and is involved in cell growth inhibition by resveratrol in lung cancer. Toxicol. Appl. Pharmacol..

[127] Cai Z.G., Aguilera F., Ramdas B. (2020). Targeting Bim via a lncRNA Morrbid regulates the survival of preleukemic and leukemic cells. Cell Rep..

[128] Zhang M.G., Yu X., Zhang Q.Y. (2022). MIR4435-2HG: a newly proposed lncRNA in human cancer. Biomed. Pharmacother..

[129] Zhao F.N., Liu Y.L., Tan F.S. (2022). MIR4435-2HG: a tumor-associated long non-coding RNA. Curr. Pharmaceut. Des..

[130] Lv C.W., Yu H.F., Wang K.Y. (2022). ENO2 promotes colorectal cancer metastasis by interacting with the LncRNA CYTOR and activating YAP1-induced EMT. Cells.

[131] Zhu H.Z., Shan Y.Q., Ge K. (2020). LncRNA CYTOR promotes pancreatic cancer cell proliferation and migration by sponging miR-205-5p. Pancreatology.

[132] Li J.Z., Sun K., Zhang M.M. (2023). Long non-coding RNA CYTOR enhances gastric carcinoma proliferation, migration and invasion via the miR-136-5p/HOXC10 axis. Am. J. Cancer Res..

[133] Wang D., Zhu X.J., Siqin B. (2022). Long non-coding RNA CYTOR modulates cancer progression through miR-136-5p/MAT2B axis in renal cell carcinoma. Toxicol. Appl. Pharmacol..

[134] Yu J.P., Shen T.Y., Li Y. (2023). CYTOR drives prostate cancer progression via facilitating AR-V7 generation and its oncogenic signalling. Clin. Transl. Med..

[135] Ou C.L., He X.Y., Liu Y. (2023). lncRNA cytoskeleton regulator RNA (CYTOR): diverse functions in metabolism, inflammation and tumorigenesis, and potential applications in precision oncology. Genes Dis..

